# Extracellular vesicles with diagnostic and therapeutic potential for prion diseases

**DOI:** 10.1007/s00441-022-03621-0

**Published:** 2022-04-08

**Authors:** Arun Khadka, Jereme G. Spiers, Lesley Cheng, Andrew F. Hill

**Affiliations:** 1 Department of Biochemistry & Chemistry, La Trobe Institute for Molecular Science, La Trobe University, Bundoora, VIC 3086 Australia; 2 Institute for Health and Sport, Victoria University, Footscray, VIC Australia

**Keywords:** Prion disease, Extracellular vesicle, Neurotoxicity

## Abstract

Prion diseases (PrD) or transmissible spongiform encephalopathies (TSE) are invariably fatal and pathogenic neurodegenerative disorders caused by the self-propagated misfolding of cellular prion protein (PrP^C^) to the neurotoxic pathogenic form (PrP^TSE^) via a yet undefined but profoundly complex mechanism. Despite several decades of research on PrD, the basic understanding of where and how PrP^C^ is transformed to the misfolded, aggregation-prone and pathogenic PrP^TSE^ remains elusive. The primary clinical hallmarks of PrD include vacuolation-associated spongiform changes and PrP^TSE^ accumulation in neural tissue together with astrogliosis. The difficulty in unravelling the disease mechanisms has been related to the rare occurrence and long incubation period (over decades) followed by a very short clinical phase (few months). Additional challenge in unravelling the disease is implicated to the unique nature of the agent, its complexity and strain diversity, resulting in the heterogeneity of the clinical manifestations and potentially diverse disease mechanisms. Recent advances in tissue isolation and processing techniques have identified novel means of intercellular communication through extracellular vesicles (EVs) that contribute to PrP^TSE^ transmission in PrD. This review will comprehensively discuss PrP^TSE^ transmission and neurotoxicity, focusing on the role of EVs in disease progression, biomarker discovery and potential therapeutic agents for the treatment of PrD.

## Introduction

Prion diseases (PrD) or transmissible spongiform encephalopathies (TSE) are progressively rapid and fatal neurodegenerative diseases (NDs) with a defining hallmark of vacuolation in the brain tissue (Besnoit and Morel 1898). Similar to other NDs with specific protein misfolding–related neurodegeneration, PrD consists of a pathogenic form (PrP^TSE^) of normal cellular prion protein (PrP^C^) (Prusiner 1998). However, unlike other NDs, the unique interspecies transmissibility and pathogenic nature of PrD make PrD more hazardous (Beck et al. 1969; Gajdusek et al. 1966; Gibbs et al. 1968). There are various forms of animal and human prion diseases, with one of the highly studied animal PrD being bovine spongiform encephalopathy (BSE; also known as ‘mad cow disease’) in cattle which was discovered in the UK for the first time in 1984–1985 (Wells et al. 1987). Soon after the UK BSE outbreak, a new form of human prion disease emerged with distinct pathophysiology termed variant CJD (vCJD) (Will et al. 1996). Thus, the spread of vCJD has been associated with the consumption of BSE-infected meat products. Other animal prion diseases include transmissible mink encephalopathy (TME) in mink, chronic wasting disease (CWD) in cervids, feline spongiform encephalopathy in domestic cats and exotic ungulate encephalopathy (EUE) in Nyala, greater kudu and oryx. A new animal prion disease in the dromedary camel of Algeria has been detected with different biochemical properties of PK-resistant prion protein to that of BSE and scrapie (Babelhadj et al. 2018).

Human PrD are differentiated into three types based on aetiologies which include genetic, sporadic and acquired. PrD, which fall under the genetic aetiologies due to point mutations or octapeptide repeat insertions in the *PRNP* gene, includes familial CJD (fCJD), Gerstmann–Stäussler–Scheinker (GSS) and fatal familial insomnia (FFI) while sporadic CJD (sCJD) presents with disease aetiology from unknown origins. PrD with acquired aetiologies from exposure to pathogenic prions includes iatrogenic CJD (iCJD), vCJD, and kuru. To date, various human-to-human iCJD transmissions occurred due to incomplete decontamination of surgical equipment, corneal grafting, dura mater grafting, cadaveric pituitary-derived growth hormone or gonadotrophin, and blood transfusions (Brown et al. 2006). The approximate prevalence of human PrD equates to 85–90% for sCJD cases, 10% for fCJD cases and less than 2–5% for acquired CJD (Chen and Dong 2016). Although the acquired CJD cases are of lower prevalence, the experimental disease transmissibility rate from humans to primates was highest in acquired CJD compared to sCJD and fCJD (Brown et al. 1994).

## Protein-only hypothesis of prion replication

**Fig. 1:**
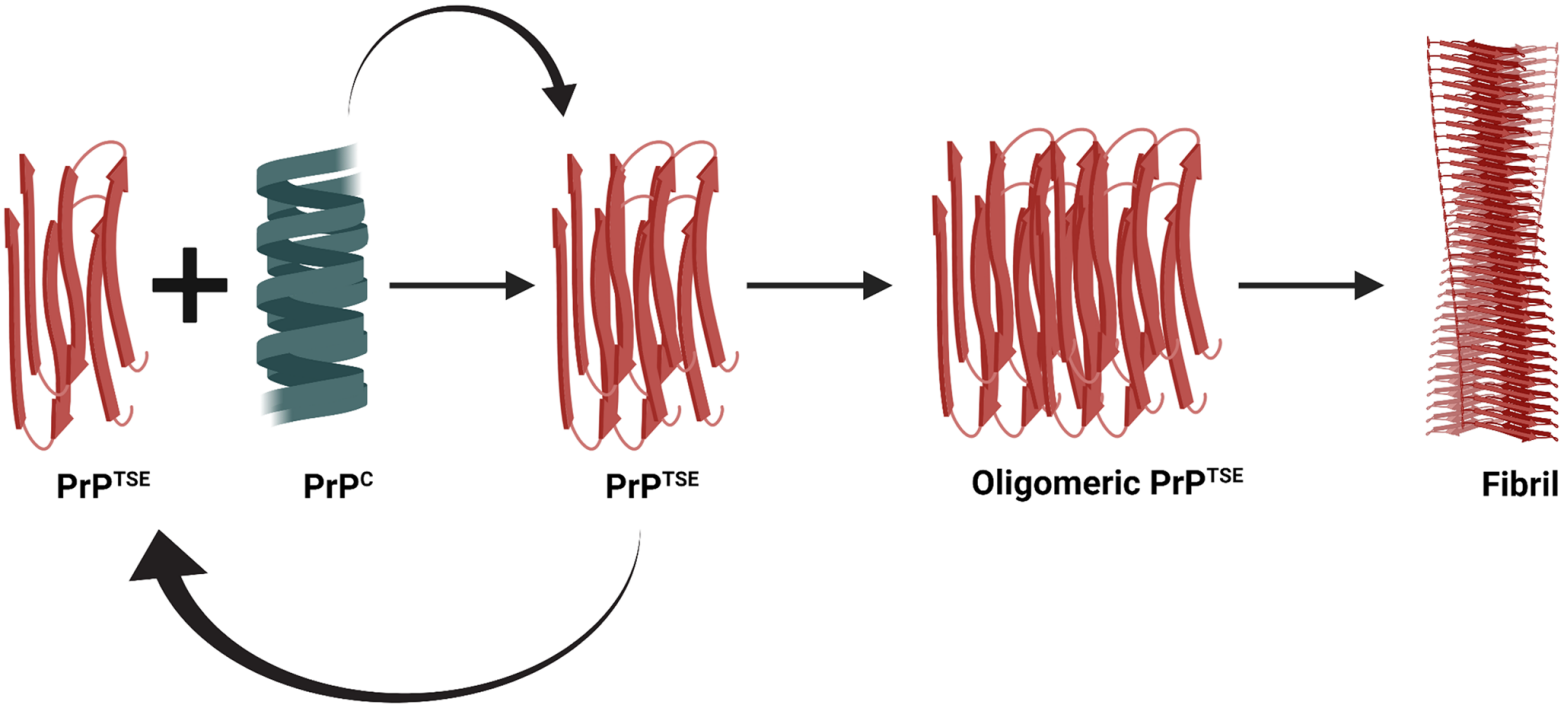
Schematic diagram illustrating the protein-only hypothesis. Here, the pathogenic form of prion protein (PrP^TSE^) is the sole causative agent of the disease which acts as a seed in the cyclic conversion of normal cellular prion (PrP^C^) into the misfolded pathogenic isoform (PrP^TSE^) in an autocatalytic fashion. These newly formed PrP^TSE^ further acts as a seed to this conversion mechanism to form PrP^TSE^ oligomers and fibrils. This figure was generated using Biorender

Extensive purification of the scrapie prion protein with subsequent sodium dodecyl sulphate (SDS) denaturation, proteinase K (PK) treatment and high-performance liquid chromatography (HPLC) purification yielded the significant component of a single protein ranging 27–30 kDa in size (PrP 27–30) (Prusiner et al. 1984). This purified PrP27–30 amino acid sequencing and the direct amino acid sequencing of the purified scrapie rods produced identical 15 N-terminal amino acid residues indicating that scrapie rods are a polymer of identical proteins. Next, the same gene encoded the healthy and disease-associated forms of prion protein in both human and mouse (Basler et al. 1986, Oesch et al. 1985). Moreover, a single uninterrupted coding gene for the prion protein suggests that the difference in healthy and scrapie associated prion protein could arise from post-translational modifications, sequence variations or conformational changes, not due to an alternative splicing mechanism (Basler et al. 1986). Subsequent experiments transferring mouse scrapie prions into mice devoid of prion protein demonstrated that these did not develop the disease and showed no behavioural changes (Bueler et al. 1993). All these attempts to determine the infectious nature of PrD led to the formation of the protein-only hypothesis, which considers the PrP^TSE^ isoform of the normal PrP^C^ protein as the sole causative agent of disease transmission (Prusiner 1998) (Fig. 1). The generation of the PrP^TSE^ isoform is either due to a gene mutation or via exogenous PrP^TSE^ exposure, and PrP^TSE^ would subsequently seed propagating the conversion of PrP^C^ into PrP^TSE^ in an autocatalytic fashion.

## PrP^C^ structure, biogenesis and physiological function

PrP^C^ has high endogenous expression in neurons and is encoded by only one exon out of three exons in the PrP^C^ gene at chromosome 20 (Basler et al. 1986). The 253 amino acid sequence of prion protein contains a signal peptide in the first 22 amino acids, which is cleaved once it reaches the ER. PrP^C^ undergoes various post-translational modifications, which add a glycosylphosphatidylinositol (GPI) anchor at the C terminus (residue 230) and two N-linked glycans at residues 181 and 197. Nuclear magnetic resonance (NMR) examination of PrP^C^ structure revealed an intrinsically disordered N-terminal tail (residues 23–128), three α-helical regions (two of them linked by a disulphide bridge) and a short anti-parallel β-sheet (Riek et al. 1997). The N-terminal region contains four identical, highly conserved octapeptide repeats (residues 51–91) (Kim et al. 2008). In the mature form, PrP^C^ is translocated to the outer leaflet of the plasma membrane in lipid raft regions and a significant portion to the non-raft regions (Sarnataro et al. 2004; Sunyach et al. 2003). The cell surface PrP^C^ is rapidly endocytosed and recycled back via coated pits (Shyng et al. 1994; Sunyach et al. 2003). In the cytosol, most PrP^C^ is found in the multivesicular bodies (MVBs), where it is packaged in extracellular vesicles (EVs; endosomal derived exosomes) and released into the extracellular environment (Guo et al. 2015; Mironov et al. 2003; Yim et al. 2015).

Although early experiments showed PrP^C^ knock-out mice exhibited no significant changes in behaviour and phenotype, a growing number of cell culture–based models attribute multimeric functions to PrP^C^ (Büeler et al. 1992). Using a mass spectrometry approach, Zafar and colleagues (2011) showed many PrP^C^ binding partners with primary predicted functions, including cell growth, signal transduction, cellular metabolism and stress pathways (Zafar et al. 2011). In addition, PrP^C^ maintains neuronal development (Steele et al. 2006), neurite outgrowth (Santuccione et al. 2005), synapse health (Laurén et al. 2009; Šišková et al. 2013), myelin maintenance (Bremer et al. 2010; Küffer et al. 2016), cellular metal ion homeostasis (Pauly and Harris 1998; Thompsett et al. 2005; Watt et al. 2012), circadian rhythm regulation (Huber et al. 2002; Tobler et al. 1996), protection from stress (Rachidi et al. 2003; Zanata et al. 2002) and neural cell adhesion molecule 1 (NCAM 1) deregulations (Mehrabian et al. 2016; Schmitt-Ulms et al. 2001). Most recently, the N-terminal copper-binding site of PrP^C^ was demonstrated to be essential for neuronal protection and neuritogenesis (Nguyen et al. 2019). In addition, to overcome the issue with genetic artefacts associated to lack of proper PrP^C^ knockout models in several of the PrP^C^ functions indicated above, a more physiologically relevant co-isogenic *Prnp*^*0/0*^ mouse model (*Prnp*^*ZH3/ZH3*^) was developed (Matamoros-Angles et al. 2022; Nuvolone et al. 2016). The co-isogenic PrP^C^ knockout model studies demonstrated a vital role in peripheral myelin maintenance, neural network formations, synaptic regulations and cognitive abilities (Matamoros-Angles et al. 2022; Nuvolone et al. 2016). Thus, multiple functions of PrP^C^ suggest that the abnormal conformation due to conversion into PrP^TSE^ generates potential disturbances in various biological processes leading to pathogenesis in PrD.

## Structure of PrP^TSE^

To elucidate the nature of PrP^TSE^ and its autocatalytic propagation, it is essential to understand the structure of PrP^TSE^. Unlike PrP^C^, the aggregated, insoluble and variably post-translationally modified nature of PrP^TSE^ has hampered high-resolution structural identification. Early biophysical analysis demonstrated that PrP^TSE^ possesses an increased β-sheet content and reduced α-helix content to PrP^C^ (Caughey et al. 1991). Further advancement on the structural analysis of PrP^TSE^ includes electron microscopy of N-terminally truncated isomorphous 2D scrapie prion crystals (Wille et al. 2002) and the refinement in modelling of this structure (Govaerts et al. 2004). Together, these studies predicted that the amyloids contain the trimeric parallel left-handed β-sheet structure of PrP^TSE^***.*** The previously predicted model incorporated the α-helix from the PrP^C^ structure, which was ruled out with hydrogen–deuterium exchange-coupled mass spectrometry analysis of mammalian PrP^TSE^ (Smirnovas et al. 2011). Although consistent with the previous electron crystallography study, the PrP^TSE^ core consisted of β-sheet structure with short turns and/or loops.

Subsequent studies using transgenic mice expressing mammalian GPI-less PrP^TSE^ exhibiting limited proteolysis with proteinase K coupled mass spectrometry predicted the four-rung β-solenoid (4RβS) model (Silva et al. 2015; Vazquez-Fernandez et al. 2012). The 4RβS model consists of proteinase K–sensitive, flexible loops or stretches that connect the highly compact β-strand solenoid core. This 4RβS core of PrP^TSE^ originally came from the X-ray fibre diffraction experiment of infectious prions (Wille et al. 2009). Recent support of the predicted 4RβS model of PrP^TSE^ came from the electron cryomicroscopy analysis of GPI-less amyloid fibril, demonstrating an average fibril repeating unit of 19.1 Å (Vazquez-Fernandez et al. 2016). Next, a comprehensive atomistic analysis using experimental and computational findings presented a more physically plausible model supporting 4RβS and with the stability equivalent to a naturally occurring β-solenoid protein (Spagnolli et al. 2019). The simulations performed in this study constructed an underlying mechanism of PrP^TSE^ autocatalysis, which proposed a sequential addition of rungs where the C-terminal rung of the β-solenoid acts as a template in the conversion of PrP^C^ to PrP^TSE^. Strengthening the 4RβS model of PrP^TSE^, isolation of infectious BSE prions for electron microscopy experiments and three-dimensional construction analysis showed rod-shaped fibrillar morphology with configuration aligning to that of the compact 4RβS model (Kamali-Jamil et al. 2021). While most amyloid studies support the four-rung β-solenoid model, other studies using solid-state NMR and side-directed spin labelling in recombinant amyloid fibrils demonstrated that PrP^TSE^ fibrils exhibit parallel-in-register intermolecular β-sheet (PIRIBS)–based structure (Cobb et al. 2007; Groveman et al. 2014; Tycko et al. 2010). A recent study supporting PIRIBS model with near atomic resolutions from electron cryomicroscopy was obtained from fully infectious scrapie prion isolates (Kraus et al. 2021). To better solve the structure of PrP^TSE^, both models should be considered to incorporate PrP^TSE^ complexity regarding different conformations and infectivity levels.

## Prion strain variation and species barriers

The numerous types of prion disease find their origins in different strains of the pathogenic prion protein that exhibit different biochemical profiles, incubation times and distribution of brain PrP^TSE^ lesions. A new form of prion strain can be established when the PrP^TSE^ from one animal species is transmitted and serially passaged to a different species (Kimberlin and Walker 1978). Primarily, strain tropism is shaped from the host PrP^C^ gene sequence and other possible host environmental factors such as lipids, RNA, chaperones and glycosaminoglycans (Baron and Caughey 2003; Geoghegan et al. 2007). Prion disease transmission from one animal species to another can have a varying degree of species barrier based on the efficiency of disease propagation and onset of clinical symptoms, which has been linked to the prion strain differences. A classic example of this is the successful infection of transgenic mice expressing human prions but not the wild-type mice when infected with human sCJD prion seeds (Collinge et al. 1995).

**Fig. 2:**
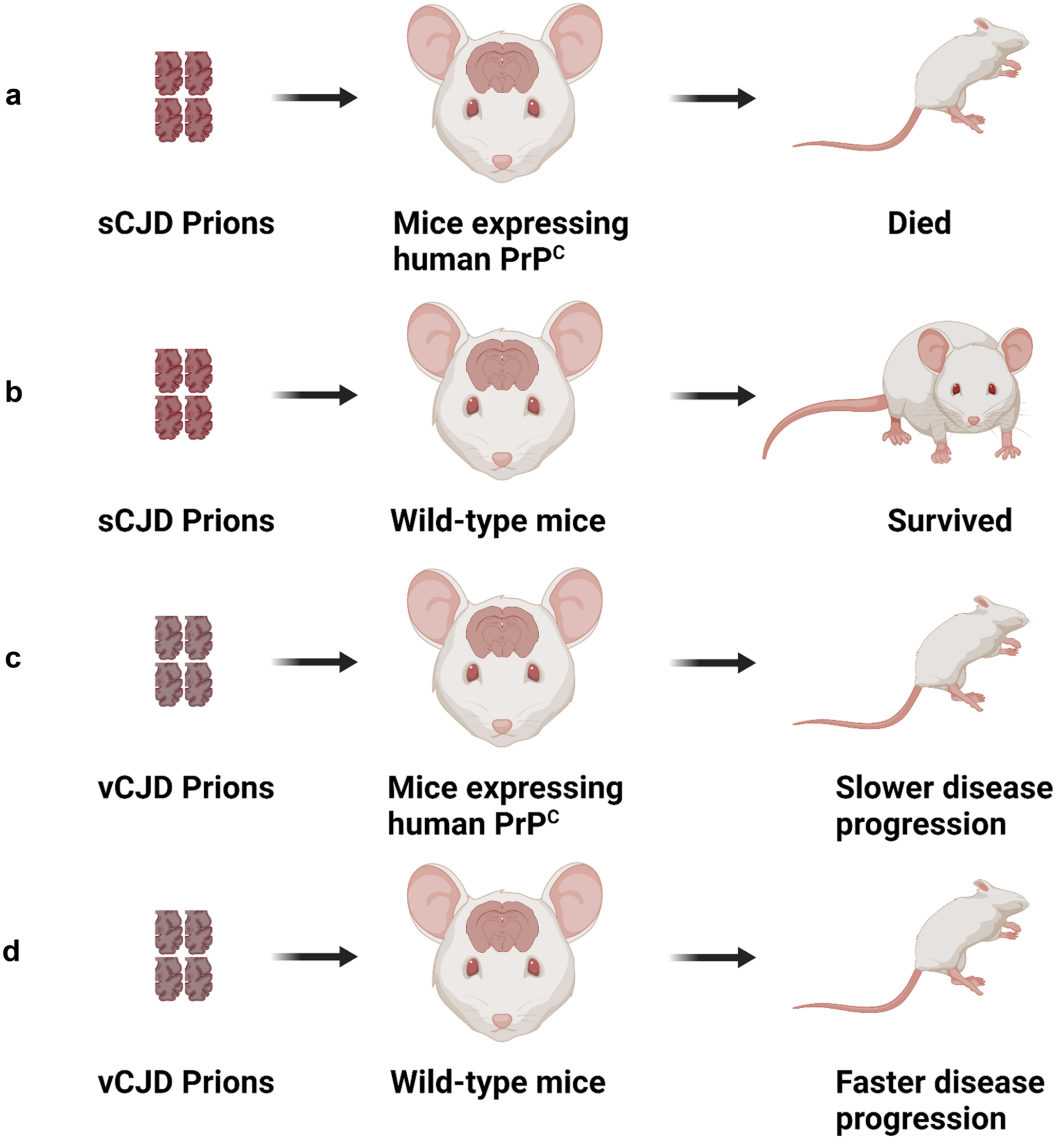
Schematic diagram illustrating the classical model of species barrier associated strain differences. Only mice expressing human cellular prion protein (PrP^C^) succumb to disease, while wild-type mice remain resistant when injected with sporadic CJD (sCJD) prion strain. However, infection with a different prion strain of variant CJD (vCJD) is capable of bypassing the species barrier, and wild-type mice succumb to the disease. This figure was generated using Biorender

Conversely, vCJD prions successfully infected mice PrP^C^ more efficiently than transgenic mice expressing only human PrP^C^ (Collinge et al. 1996) (Fig. 2). Later, it was discovered that transgenic mice expressing human PrP^C^ with codon 129 methionine homozygosity were necessary for causing vCJD disease phenotype and circumventing the species barrier (Wadsworth et al. 2004). In vitro studies demonstrated that species barriers could be bypassed by single amino acid residue substitutions in a critical region of the PrP^C^ sequence that removes the variations between two species (Jones and Surewicz 2005; Vanik et al. 2004). More importantly, another in vitro study demonstrated that the differences in secondary structures of PrP^TSE^ fibrils result in a species barrier, with new strains bypassing the species barrier inheriting the secondary structure and morphology of the seed fibril (Jones and Surewicz 2005). This implies that the different conformations of PrP^TSE^ are transmitted as different quaternary structures to form the basis of the species barrier. A novel study utilising asymmetric-flow field-flow fractionation (AF4) isolation of infectious prions from different prions strains for dynamic and multi-angle light scattering (DLS/MALS) analysis showed quaternary structure difference resulting in PrP^TSE^ strain heterogeneity (Cortez et al. 2021).

Strain-specific tropism produces various clinicopathological features and regional specific PrP^TSE^ deposition in addition to variation in incubation time and disease duration. In terms of M1000 and MU02 prion strains, which are mouse adapted human prion strains of familial and sporadic origins, respectively, both the thalamus and hippocampus demonstrated spongiform changes. At the same time, PrP^TSE^ plaques were present only in thalamus for MU02 (Lawson et al. 2008). The molecular basis of strain tropism-related unique disease variables has been associated with the different conformations of PrP^TSE^. Moreover, differences in the clinicopathological features between two familial forms of PrD, which included widespread spongiform degeneration in fCJD and selective thalamic nuclei atrophy FFI, resulted from different PrP^TSE^ isoforms determined by a codon 129 methionine/valine (M/V) polymorphism (Goldfarb et al. 1992). Similarly, in terms of distinct pathology of sCJD, the associated variables were demonstrated to be a M/V polymorphism at codon 129 of *PRNP* as well as different PK cleavage profiles of protease-resistant core fragments (PrP^res^) (Hill et al. 2003; Kobayashi et al. 2007; Parchi et al. 2009). Based on the migration of the unglycosylated PK resistant (PrP^res^) fragment on SDS-PAGE gels, three different PrP^res^ subtypes have been identified, which include type 1 (21 kDa), type 2 (19 kDa) and type intermediate (i;20 kDa) (Parchi et al. 2009). Recently, a study demonstrated the involvement of the PrP^C^ GPI anchor in determining the species barrier by detecting increased infectivity of GPI-deficient mouse scrapie PrP^TSE^ to the tg44 mice overexpressing human PrP^C^ (Race et al. 2015). Although structural differences of PrP^TSE^ have been proven to be the primary source of the species barrier, other host environmental factors should be considered for having a crucial role in transferring infectivity.

Protein misfolding cyclic amplification (PMCA) is a technique that converts normal brain homogenate PrP^C^ substrate into pathogenic PrP^TSE^ form using multiple sonication/incubations in the presence of a PrP^TSE^ seed. It is concerning that strain-specific interspecies barrier-crossover was detected with PMCA when stabilised or adapted cervid PrP^TSE^ seed successfully converted transgenic mice overexpressing human PrP^C^ substrate (Barria et al. 2011). Successful adaptation of this natural source–extracted cervid PrP^TSE^ seed was performed with one or two passages in a PMCA experiment using mice overexpressing cervid PrP^C^ as a substrate and in an in vivo experiment using mice overexpressing cervid PrP^C^. In addition, other studies involving strain adaptation using in vivo models and PMCA experiments demonstrated that the adapted strain had increased infectivity in subsequent passaging to the adaptation species (Barria et al. 2011; Chianini et al. 2012). However, one recent study demonstrated the existence of a non-adaptive form of prion replication using in vivo models and PMCA, with transgenic mice expressing horse and deer PrP^C^ retaining strain properties of source PrP^TSE^ (Bian et al. 2017). Overall, it is essential to note that the interspecies barrier potentially depends both on the strain types and the degree of the strain adaptation.

## PrP^TSE^ and neurotoxicity

Even though neuronal loss and PrP^TSE^ deposition are the characteristic features of prion-infected brains, the mechanism of neurotoxicity has remained elusive. However, several studies have investigated the synergistic effect generated by the interaction of PrP^C^ and PrP^TSE^, providing some explanation on the neurotoxic effects of PrP^TSE^. Grafting neural tissue overexpressing PrP^C^ into PrP^C^ null mice brains and intracerebrally injecting scrapie prions produced severe histopathological changes exclusively in the grafted tissue (Brandner et al. 1996). Significant amounts of PrP^TSE^ were further detected in the surrounding brain regions devoid of PrP^C^ which failed to elicit histopathological changes even after 16 months. The discovery that PrP^C^ expression in the host species was directly proportional to the clinical onset of the disease suggested that the PrP^C^/PrP^TSE^ interaction catalyses the production of neurotoxic species (Sandberg et al. 2011). Moreover, a growing body of evidence suggests cell membrane PrP^C^ elicits downstream neurotoxicity following its interaction with PrP^TSE^ (Altmeppen et al. 2015; Herrmann et al. 2015; Resenberger et al. 2011a).

It has been demonstrated that the small oligomeric unit of PrP with a mass equivalent to 14–28 PrP units exhibits more infectivity than higher fibrillar amyloids (Silveira et al. 2005). Further studies demonstrated that the small soluble PrP^TSE^ oligomers are also the agents of neurotoxicity (Kazlauskaite et al. 2005; Minaki et al. 2009; Sandberg et al. 2011; Simoneau et al. 2007). Early neurotoxic molecular disturbances have been implicated in the impairment of the ubiquitin/proteasome system (Deriziotis et al. 2011; Kristiansen et al. 2007; McKinnon et al. 2016; Thibaudeau et al. 2018) and persistent translational repression in global proteins by activating the unfolded protein response (UPR) through increased phosphorylated translation initiation factor 2α (eIF2α-P) (Halliday et al. 2015; Moreno et al. 2012). Moreover, several studies show impairment of autophagy as a mechanism of neurotoxicity in PrD, with the enhancement of autophagy promoting clearance of intracellular PrP^TSE^ deposits (Cortes et al. 2012; Thellung et al. 2018; Xu et al. 2012). Abnormal autophagic activation and neuronal apoptosis were also observed following treatment with highly toxic misfolded prion protein, with the underlying mechanisms including severe nicotinamide adenine dinucleotide (NAD^+^) starvation followed by ATP depletion (Zhou et al. 2015). In addition, p38 mitogen-activated protein kinase (MAPK) synaptic degeneration–associated neurotoxicity mechanisms were detected upon treating hippocampal neurons with PrP^TSE^. An inhibition of this pathway reversed the neurodegeneration process (Fang et al. 2018). A very recent study with an in vivo spontaneous model of TSE showed a pathogenic mechanism that accounted for endoplasmic reticulum and proteasomal stress with elevated levels of protein kinase RNA-like endoplasmic reticulum kinase (PERK), immunoglobulin heavy chain-binding protein (BiP), protein disulphide isomerase (PDI) and ubiquitin at both preclinical and clinical mice (Otero et al. 2021). Together, this indicates that the neurodegenerative processes of PrP^TSE^ extend beyond misfolded protein aggregation and include dysregulated cellular metabolism, signalling pathways and proteostasis mechanisms, highlighting the multifaceted nature of PrD.

## PrP^TSE^ conversion and trafficking

The molecular mechanisms governing the autocatalytic conversion of PrP^C^ into PrP^TSE^ are still unclear. Hydrogen–deuterium exchange-coupled mass spectrometry demonstrated the region spanning residues from 80 to 90 to the C-terminal end undergoing refolding during the conversion of PrP^C^ into PrP^TSE^ (Smirnovas et al. 2011). Early insights into this process suggested the conversion of PrP^C^ into an intermediate product (PrP*) (Cohen et al. 1994) or second product by redox reactions to cross the energy barrier for PrP^TSE^ formation (Lee and Eisenberg 2003). PMCA demonstrated that co-factor molecules like RNA or phosphatidylethanolamine (PE) play crucial roles in regulating the conformation, infectivity and strain properties of PrP^TSE^ (Deleault et al. 2012; Supattapone 2014). A recent study using PMCA supported the critical role of cofactors in the conversion process, which required glycosaminoglycans (GAGs), including heparan sulphate and the analogue, heparin (Imamura et al. 2016). Altogether, these studies suggest that the refolding of PrP^C^ in the presence of seed PrP^TSE^ happens at the C-terminal region of PrP^C^ and requires environmental host factors or cofactors assisting the process.

Mechanisms of PrP^TSE^ uptake are poorly understood due to the difficulty in differentiating PrP^TSE^ from PrP^C^. A growing body of in vitro studies examining major interacting partners demonstrated that laminin receptor protein (LRP) (Gauczynski et al. 2006) and low-density lipoprotein receptor-related protein (LRP1) (Jen et al. 2010) may take part in PrP^TSE^ uptake, whereas PrP^C^ (Greil et al. 2008) and GAGs (Wolf et al. 2015) may not be crucial for this process. A recent study demonstrated that internalisation of PrP^TSE^ is followed by trafficking through the endo-lysosomal pathway and endocytic recycling complex (Yamasaki et al. 2014). Lysosomes have been designated as the significant disintegration site of the internalised PrP^TSE^ (Choi et al. 2014; Krejciova et al. 2014). The detection of nascent formed PrP^TSE^ residing in the amyloidogenic fibrillar strings on the cell surface suggested a potential role of the plasma membrane during PrP^TSE^ trafficking and conversion (Rouvinski et al. 2014). Another study using immunofluorescence and electron microscopy supported the plasma membrane and endolysosomal compartments as potential conversion sites, with extracellular trafficking occurring via exosomes, a specific subclass of EVs (Veith et al. 2009). A subsequent study demonstrated the reduction and increase of PrP^TSE^ content when blocking MVB maturation and enhancing cargo recycling through the MVB, respectively (Yim et al. 2015). Experiments involving inhibition of the neutral sphingomyelinase pathway and stimulating exosome production demonstrated decreases in intercellular PrP^TSE^ infectivity and increased PrP^TSE^ content, respectively (Guo et al. 2016). Together, these studies indicate that the MVB could be a promising PrP^TSE^ conversion site, and exosomes could play a significant role in intercellular infectivity promoting the propagation of the disease.

## PrP^TSE^ spread throughout the body

The various aetiologies of PrD range from eating BSE-infected meat products in vCJD to corneal grafting or blood transfusion in iCJD, indicating that PrP^TSE^ spreads from the periphery to the brain. Following intraperitoneal injection of mice with scrapie prion, infection was initially found in the lymphoreticular system. The spleen was the major accumulation site before reaching the brain (Dickinson and Fraser 1969a, 1969b). Moreover, in many cases, PrP^TSE^ has been found to accumulate in the follicular dendritic cells of lymphoid tissues (Jeffrey et al. 2000; Kitamoto et al. 1991). Subsequent experiments involving functional loss or inactivation of follicular dendritic cells resulted in the delay of neuroinvasion as well as decreased susceptibility to scrapie (Mabbott et al. 2000, 2003). Recently, intracerebral inoculation of interspecies PrP^TSE^ in ovine and human transgenic mice demonstrated preferential PrP^TSE^ replication in splenic versus neuronal tissues (Beringue et al. 2012).

**Fig. 3:**
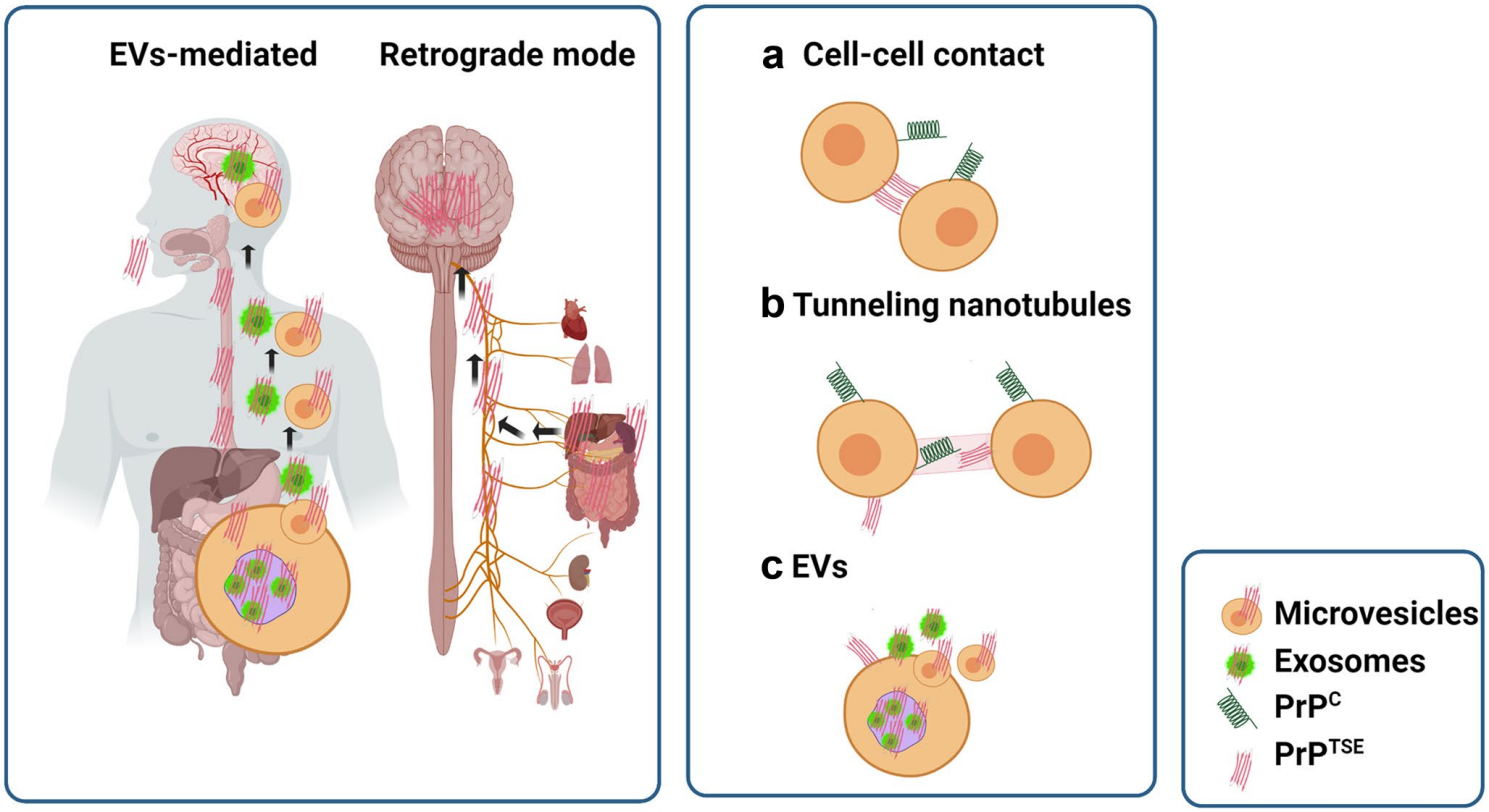
Schematic diagram showing different mechanisms of prion spread. Possible mechanisms of pathogenic prion protein (PrP^TSE^) spread to the brain from distal organs include extracellular vesicle (EV)–mediated transfer (1) or retrograde transfer across vagus and splanchnic nerves (2). The intercellular PrP^TSE^ transfer involves cell–cell contact **a**, tunnelling nanotubules **b** and EVs **c**. This figure was generated using Biorender

However, the mechanisms facilitating PrP^TSE^ spread from different regions of the body to the brain remain largely unknown. One potential route supported by immunohistochemical evidence suggests that PrP^TSE^ undergoes retrograde transmission in vagus and splanchnic nerves (McBride et al. 2001). In addition, the molecular mechanism underlying the intercellular prion spread has been proposed to primarily involve (1) direct cell-to-cell contact (Kanu et al. 2002; Paquet et al. 2007), (2) tunnelling nanotubes (Gousset et al. 2009; Langevin et al. 2010), (3) GPI painting (Baron et al. 2002) and (4) extracellular vesicles (EVs) (Fevrier et al. 2004; Guo et al. 2016; Mattei et al. 2009; Yim et al. 2015) (Fig. 3).

## EVs and their association with PrP^C^ and PrP^TSE^

**Fig. 4:**
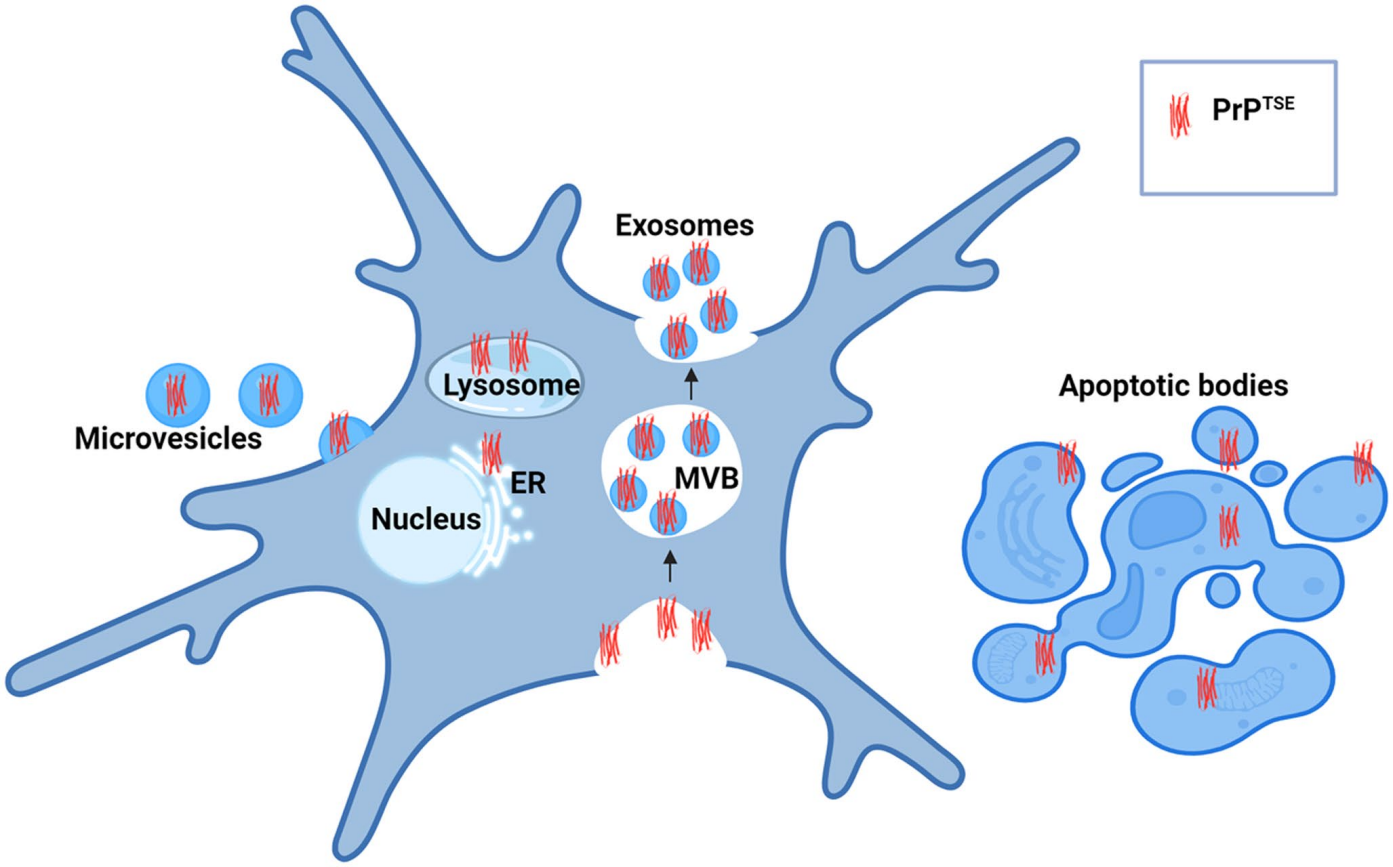
Schematic diagram showing extracellular vesicle (EV) generation and pathogenic prion protein (PrP^TSE^) localisation in EVs. Small EVs (exosomes; < 200 nm) are generated via endosomal pathways when the multivesicular bodies (MVBs) fuse with the plasma membrane. These carry several classes of marker protein including tetraspanins, endosomal sorting complex required for transport (ESCRT) proteins, neutral sphingomyelinase 2 (nSMase2), heat shock proteins (HSPs) and integrins as defined in the Minimal Information for Studies of Extracellular Vesicles (MISEV) guidelines (Théry et al. 2018). Microvesicles are generated via blebbing or shedding from the plasma membrane, and apoptotic bodies are generated via cellular fragmentation in cells undergoing apoptotic cell death. Studies have demonstrated PrP^TSE^ localisation in the plasma membrane, endolysosomal system including the endoplasmic reticulum (ER), and EVs

EVs are small and non-synaptic, with significant biological and pathological functions. They carry biological macromolecules, including proteins and genomic material, from cells through the extracellular fluids. Both eukaryotic and prokaryotic cells produce EVs. These are secreted by all cell types in the nervous system, becoming increasingly studied as a new method of intercellular communication and as a source of disease biomarkers. EVs include apoptotic bodies, microvesicles (also called ectosomes) and exosomes classified based on biogenetic pathway and size. Apoptotic bodies (500–5000 nm) arise from the cell fragmentation during apoptosis, microvesicles (200–1000 nm) from outward budding and shedding from the plasma membrane, and exosomes (40–200 nm) which are released intercellularly following production via the endosomal pathway and fusion of the MVB to the plasma membrane (Kalra et al. 2012; Thery et al. 2009) (Fig. 4).

As described above (see Structure of PrPTSE), the evidence for packaging of prion protein in the EVs provides a novel mechanism of prion-induced intercellular communication. In a recent study, EVs derived from the brain demonstrated the presence of enriched prion protein which was further increased after transient ischaemia (Brenna et al. 2020). Moreover, in the same study where brain EVs were isolated from wild-type and PrP^C^ knockout mice, EV uptake from the PrP^C^ knockout model by primary neurons, astrocytes and microglia was significantly faster and more efficient. This indicates that the association of PrP^C^ on the EVs plays a vital role in the physiological and pathophysiological conditions that could be targeted for therapeutic roles. A new study targeting astroglial communication and the movement of EVs along the neuronal surface revealed that a hydrophilic interaction of EV prion protein and neuronal prion protein is required for this movement in the majority of EVs (D'Arrigo et al. 2021). This association of EVs and prion protein for the neuron surface movement reveals the importance in intercellular communication where EVs can move across the axonal surface to reach the target sites. In addition, PrP^C^-associated EVs isolated from human plasma promote anti-inflammatory effects on LPS-induced macrophages (Mantuano et al. 2022). These studies collectively reveal a multifunctional role of PrP^C^-associated EVs in processes including EV uptake mechanisms, movements and the intercellular communications. Thus, the conversion of PrP^C^ into PrP^TSE^ would suggest a significant impact on EVs’ biophysical activities for intercellular communications.

Initial studies demonstrating exosomes could carry PrP^TSE^ capable of infectivity transfer was shown in cell model isolated exosomes with immunogold labelling on guanidium-treated samples to specifically probe PrP^TSE^ (Fevrier et al. 2004; Leblanc et al. 2006). A subsequent study isolated exosomes from infected neuronal cells to demonstrate in vitro and in vivo PrP^TSE^ association with exosomes and the ability of these to transfer infectivity (Vella et al. 2007). The isolated exosomes containing PrP^TSE^ were shown to transfer infectivity to healthy neuronal cells and non-neuronal cells in culture, indicating that infectivity transfer is possible across different cell types. Moreover, the inoculation of mice with these exosomes containing PrP^TSE^ resulted in the development of clinical prion disease. While EVs are present in all kinds of biofluids, detection of prion infectivity in the blood (Concha-Marambio et al. 2016), milk (Gough et al. 2009; Maddison et al. 2009), urine (Gonzalez-Romero et al. 2008), cerebrospinal fluid (Foutz et al. 2017; Murayama et al. 2014), and EVs isolated from plasma (Cervenakova et al. 2016; Saá et al. 2014) suggests that EVs could be a significant route of disease spread, travelling great distances within the body. Very recently in cases of sCJD, PrP^TSE^ was found to accumulate through regions of the eye, suggesting that tear EVs in prion-infected patients could also potentially carry prion infectivity (Orrù et al. 2018). Using in vitro models, cellular prion infectivity and exosomal release of prion infectivity were found to be associated with the endosomal sorting complexes required for transport (ESCRT)-dependent and -independent pathways of exosome release (Vilette et al. 2015). In addition, the transgenic RK13 cell line expressing ovine, mouse and vole PrP^C^ was used to show different prion strains that exhibit specific differential release of PrP^TSE^ associated with exosomes (Arellano-Anaya et al. 2015). This study demonstrated that most prion infectivity was associated with the exosomes from the prion-infected conditioned media, and ovine 127S infected RK13 secreted 20- to 40-fold more PrPTSE in exosomes than exosomes murine 22L and vole prions. Together, these studies indicate that EVs may be a significant source of prion infectivity and highlight how understanding the mechanisms of prion EVs’ cargo loading and cellular uptake could provide novel insight into the dysregulated biochemical functions and downstream disease processes.

## EVs as a source of biomarkers for prion disease

EVs are regarded as a source of biomarkers because of their superior properties as a storage unit for various biochemical markers like nucleic acids, proteins and lipids. Moreover, their accessibility from biofluids makes them an attractive candidate for minimally invasive diagnostic purposes. EVs extracted from the blood plasma and serum are highly enriched with microRNA (miRNA), and many have predicted targets in crucial neuronal signalling pathways. This suggests that EVs could carry brain-associated miRNAs and into the blood (Cheng et al. 2014), an idea supported by studies isolating neural-derived EVs in the blood using neural cell adhesion molecule L1 (L1CAM) which is highly expressed in the brain (Fiandaca et al. 2015; Kapogiannis et al. 2015). Further evidence of brain EVs detected in the blood includes EVs isolated from blood samples of mice that have received human glioma transplants (García-Romero et al. 2017). In addition, brain tissue–derived EV isolation can enhance the precision of diagnosis related to the affected region when correlated together with the blood-based differential miRNAs and proteins in EVs for biomarker discovery (Cheng et al. 2021).

### Selective packaging of miRNAs in EVs

Small non-coding miRNA (20–22 nucleotides long) play key roles in gene regulation either by inducing mRNA degradation or repression of mRNA translation. A plethora of miRNAs are present in the brain, indicating that complex physiological and neuronal development functions of miRNAs are more enriched in the brain regions, which shows the complex physiological functions of the brain (Sempere et al. 2004; Bak et al. 2008). miRNAs function with a dual nature consisting of divergent actions, meaning single miRNAs can target regulation of multiple genes, and/or convergent actions, where two or more miRNAs target the same untranslated region (UTR) (Lukiw and Alexandrov 2012). Many experiments have shown the involvement of miRNA in cellular stress-induced neurodegeneration together with the presence of toxic substances like reactive oxygen species (ROS) and polyglutamine (Bilen et al. 2006; Lukiw and Pogue 2007). Moreover, miRNA stability is essential for neuronal survival, with ablation of the miRNA generating enzyme Dicer in mouse Purkinje cells causing progressive loss of miRNA, resulting in slow cerebellar degeneration (Schaefer et al. 2007).

**Fig. 5:**
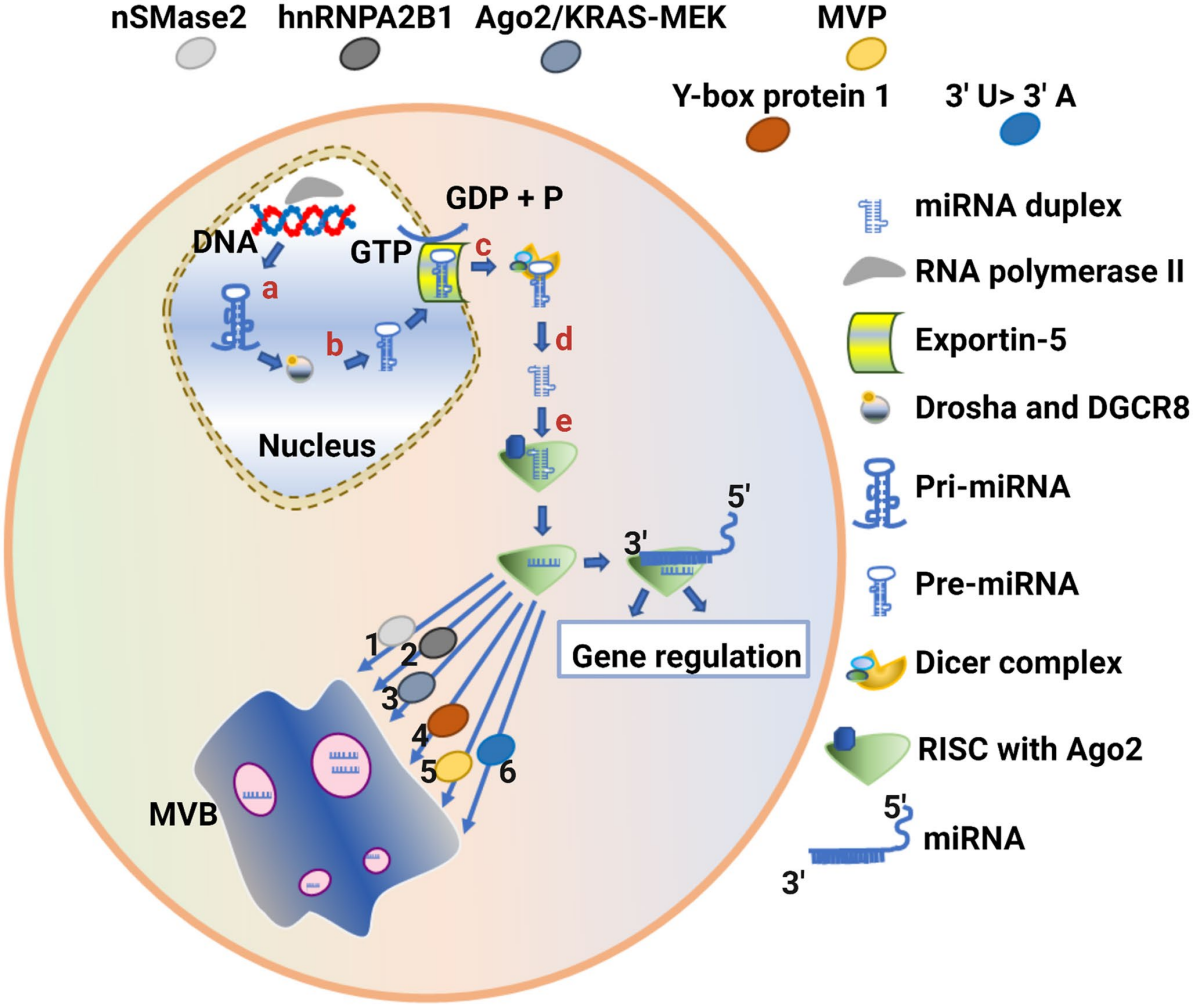
Schematic diagram showing microRNA (miRNA) biogenesis and sorting into exosomes. The alphabetical numbering shows the miRNA biogenesis pathway; **a** primary miRNA (Pri-miRNA) is synthesised in the nucleus by RNA polymerase II; **b** RNase III enzyme (Drosha) and DiGeorge syndrome critical region 8 (DGCR8) cofactor cleave Pri-miRNA into precursor miRNA (pre-miRNA); **c** pre-miRNA is exported to the cytoplasm by Exportin-5; **d** mature miRNA duplex is produced after cleavage of pre-miRNA by RNase III enzyme (Dicer); **e** RNA-induced silencing complex (RISC) produces single-stranded miRNA and transports it to the upstream mRNA sequence for its functional repression. The selective sorting of miRNA into exosomes has been demonstrated through many RNA binding proteins, including (1) neutral sphingomyelinase 2 (nSMase2), (2) heterogeneous nuclear ribonucleoprotein A2B1 (hnRNPA2B1), (3) Argonaute2 (Ago2), (4) Y-box protein 1 and (5) major vault protein (MVP). (6) The specific enrichment of 3′ uridylated (3′ U) miRNAs has been found in exosomes and 3′ adenylated (3′ A) miRNAs have been found in cells. This figure was generated using Biorender

Potential mechanisms underlying miRNA packaging into EVs have been the subject of intense scrutiny in several recent studies. Knockdown of neutral sphingomyelinase 2 (nSMase2), a regulator of ceramide biosynthesis, reduced exosomes as well as miRNA secretion, an effect that was reversed following overexpression (Kosaka et al. 2010). Furthermore, nSMase2-dependent regulation of exosomal miRNAs from cancer cells was capable of promoting angiogenesis and metastasis (Kosaka et al. 2013). However, the mechanism of nSMase2 regulation of miRNA packaging into exosomes has remained elusive. Specific sequence motifs in miRNA are now known to interact with RNA binding proteins, a mechanism that both prevents degradation and acts as a vector for cellular sorting machinery. In its sumoylated form, the heterogeneous nuclear ribonucleoprotein A2B1 (hnRNPA2B1) recognises and binds the 3′ portion of miRNA containing the GGAG motif, which controls the loading of miRNA into exosomes (Villarroya-Beltri et al. 2013). Similarly, knockout or knockdown of Argonaute2 (Ago2) in cell models reduced preferentially exported exosomal miRNAs such as miR-150, miR-100 and let-7a (Guduric-Fuchs et al. 2012). The sorting of Ago2 into exosomes was further shown to be regulated by Kirstan rat sarcoma-mitogen-activated protein kinase kinase (KRAS-MEK) signalling, with inhibition of this signalling increasing sorting of Ago2 into exosomes (McKenzie et al. 2016). The Y-box protein 1 is another candidate RNA binding protein that potentially contributes to the selective packaging of miRNA into exosomes (Shurtleff et al. 2016). Shurtleff and colleagues (2016) demonstrated that Y-box protein 1 is co-packaged with synthetic miR-223-biotin in a cell-free reaction system captured using streptavidin-coated beads and detected using streptavidin tandem-mass spectrometry. Similarly, major vault protein (MVP) was captured from exosomal lysate with a bead-based streptavidin–biotin-miR-193a system and detected with matrix-assisted laser desorption ionisation-time of flight (MALDI-TOF) mass spectrometry, suggesting that MVP binds exosomal miR-193a and may be another candidate for RNA packaging (Teng et al. 2017). These studies highlight the importance of the subcellular distribution, localisation and transport of RNA-binding proteins in the process of miRNA binding and loading into EVs. While these studies have generally focused on RNA binding proteins, there is also evidence that RNA can undergo post-transcriptional modifications, ultimately altering its functional distribution and degradation patterns (Roundtree et al. 2017). Not surprisingly, RNA sequencing identified 3′ uridylated forms of miRNA that were found enriched in exosomes while 3′ adenylated miRNA forms were enriched in cells (Koppers-Lalic et al. 2014). Together, this highlights the diversity and complexity of mechanisms required to coordinate miRNA packaging and distribution into EVs (Fig. 5).

### Potential miRNA biomarkers in prion disease

Initial attempts to determine the role of miRNA in neurodegeneration related to prion infection were performed in mouse brains infected with mouse-adapted scrapie. These experiments identified miRNAs including miR-342-3p, miR-320, let-7b, miR-328, miR-128, miR-139-5p and miR-146a that were significantly upregulated while miR-338-3p and miR-337-3p were significantly downregulated. Moreover, bioinformatics and biochemical analysis showed that these miRNAs have functions in neuronal degradation and disease progression (Saba et al. 2008). A similar study to characterise deregulation of miRNA that potentiates the pathogenesis in human PrD was performed in BSE-infected *Cynomolgus* macaques. Microarray analysis demonstrated that hsa-miR-342-3p and hsa-miR-494 were significantly upregulated in brain samples (Montag et al. 2009). More recently, a study using mice infected with 139A − , ME7 − and S15 scrapie strains found two novel miRNAs, miR-2 and miR-20, downregulated by all three prion strains in the infected brain samples (Gao et al. 2016). In the same study, 14 commonly decreased and 22 commonly increased miRNAs were detected in the brain samples of all three prion strain types. Such common deregulated miRNAs indicate similar disease pathogenesis mechanisms and highlight how miRNA could act as a potential biomarker candidate.

One of the first studies that showed the diagnostic potential of EVs miRNA was carried out in prion-infected mouse hypothalamic neuronal cell cultures (Bellingham et al. 2012). This study observed upregulation of let-7i, miR-21 and downregulation of miR-146a in both prion-infected neuronal cells and exosomes secreted by these cells, whereas let-7b, miR-128a, miR-222, miR-29b, miR-342-3p and miR-424 were significantly upregulated only in exosomes (Bellingham et al. 2012). A recent prion EV miRNA biomarker study consisted of a longitudinal analysis of miRNA on M1000 prion–infected mice thalamus and serum EVs of pre-clinical and clinical samples (Cheng et al. 2021). Three potential miRNA biomarkers, miR-1a-3p, miR-181a-5p and miR-142-3p, were deregulated in both thalamus and serum EVs. Furthermore, 17 miRNAs that were differentially expressed in mice serum EVs were validated in a second independent cohort of sCJD patient serum samples. Bioinformatics predicted that the best combination of four miRNAs as a biomarker consisted of miR-423-3p, miR-101-3p, miR-1306-5p and miR-142-3p, which predicted sCJD with an area under the curve (AUC) of 0.800 (85% sensitivity and 66.7% specificity). Another recent study of naturally infected sheep with scrapie demonstrated significant upregulation of miR-21-5p in both plasma EVs and total CSF whereas other promising miRNAs significantly upregulated in the total CSF included miR-342-3p, miR-146a-5p and miR-128-3p (López-Pérez et al. 2021). Further work is required to test the consistency and role of these EV-associated miRNAs in PrD. However, the appearance of similar miRNA across different types/strains/species of PrD is highly encouraging and the utilisation of these in combination for use as a biomarker has enormous potential as a diagnostic tool in PrD.

## Deregulated mitochondrial biomarkers in prion disease

After more than three decades of research following the discovery of the proteinaceous nature of PrD, several deregulated proteins have been identified. Despite discovering several proteins involving various strains and models of prion disease, no early disease-associated protein biomarker has yet been identified. Proteins that have shown potential as prion biomarkers include 14–3-3 and tau from CSF (Hsich et al. 1996; Otto et al. 2002). More recent studies that utilise CSF biomarkers have established that increases in neurofilament light chain and α-synuclein can differentially diagnose sCJD among various neurodegenerative diseases (Llorens et al. 2017; Zerr et al. 2018).

Across different models of PrD, the vast majority of deregulated proteins have been found with the application of mass spectrometry. Global proteomic analysis discovered that PrD-associated deregulated proteins fall into various functional subgroups, including protein folding, cell death, synaptic dysfunction, ion transport, oxidative stress, immune regulation and energy metabolism. One such study on mouse-adapted scrapie prion strain (22L)–infected N2a cells overexpressing murine prion protein demonstrated upregulation in the redox chaperone, protein disulphide isomerase (PDI) (Provansal et al. 2010). The majority (41.5%) of differentially expressed proteins identified in this study were involved in energy metabolism, including upregulation of the oxidative phosphorylation protein, ATP synthase. Another proteomics study on sCJD patient CSF samples demonstrated that the highest percentage of deregulated proteins, accounting for 22.10% of the differential expression, was involved in cellular metabolism (Wang et al. 2017). In addition, tricarboxylic acid (TCA) cycle proteins were also discovered, including succinate dehydrogenase subunit A (SDHA), aconitase 2 (ACO2) and malate dehydrogenase 2 (MDH2). Proteomic profiling of brain homogenates from C57BL/10SnJ mice infected with mouse-adapted ovine scrapie strain (RML) revealed increased dysfunction of the mitochondrial proteome, which was associated with neurodegeneration (Moore et al. 2014). This mitochondrial-associated apoptosis was related to elevated levels of Mitofilin, heat shock protein family A (Hsp70) member 9 (HSPA9), apoptosis-inducing factor 1 (AIF1) and various other calcium-regulated mitochondrial changes. One of the top pathways discovered in the first human PrD proteome analysis study identified fatty acid elongation in mitochondria, including deregulated enoyl-CoA hydratase, short chain 1 (ECHS1) (Shi et al. 2015). This brain region–specific proteomic analysis in different human PrD demonstrated higher cerebellum pathogenesis compared to the cortex region. Moreover, a recent proteomics study on sCJD patients’ cerebellum discovered reactive oxygen species (ROS) scavenger protein deglycase DJ-1 upregulation only in VV2 subtype, with upregulation at the mRNA level in both MM1 and VV2 subtypes (Tahir et al. 2018). Other mitochondrial associated proteins that were identified with this proteomic analysis included apoptosis-inducing factor mitochondria associated 1 (AIFM1), dihydrolipoamide S-succinyltransferase (DLST), translocase of outer mitochondrial membrane 70A (TOMM70A) and succinate-CoA ligase ADP-forming subunit beta (SUCLA2). Together, this suggests that mitochondrial dysfunction may be a primary driver of PrD pathophysiology, potentially underlying key metabolic differences contributing to neurodegeneration. The association of several key metabolic enzymes responsible for the generation of ATP suggests PrD likely disrupts ATP production and cellular energy homeostasis. Moreover, mitochondrial stress, particularly through the oxidative phosphorylation chain, likely results in redox dyshomeostasis and cellular oxidative stress, a key player in the neurodegenerative processes associated with PrD.

## EV mitochondrial biomarkers

Neurodegeneration related to mitochondrial dysfunction has been extensively studied in several neurodegenerative diseases, including PrD. However, investigating mitochondrial mechanisms of neuronal cell death or its biochemical and biological profiles is still lacking in PrD models compared to neurodegenerative diseases like Parkinson’s disease (PD) and Alzheimer’s disease (AD). An early study of scrapie-infected hamster brain cerebral cortex transmission electron microscopy images depicted alterations in mitochondrial morphology and depletion of mitochondrial matrix and cristae (Choi et al. 1998). In addition, the same study demonstrated a deficiency of various mitochondrial enzymes, including manganese superoxide dismutase (Mn-SOD), cytochrome *c* oxidase (complex IV) and ATPase (complex V). A more recent study validated mitochondrial respiratory chain deficiency in human sCJD brain temporal cortex with immunohistochemistry, demonstrating a loss of all mitochondrial complexes (I–V) (Flønes et al. 2020). Several further studies have investigated neuronal death linked to mitochondrial dysfunction in an in vitro model system of PrD. Early co-culture experiments using primary cortical neurons together with scrapie-infected N2a cells demonstrated mitochondrial clustering in the perinuclear region of the primary cells (Resenberger et al. 2011b). A recent study isolated mitochondrial fractions from the scrapie SMB-S15 cell model and scrapie 139A- and ME7-infected mouse brains and demonstrated increased expression of phosphatase and tensin homolog (PTEN)-induced kinase1 (PINK1) and parkin in the infected cells and tissues (Gao et al. 2020). In addition, immunohistochemical analysis of various brain sections in this study observed the activation of mitophagy by PrP^TSE^ through the colocalisation of PINK1 and parkin with PrP^TSE^. As noted above, this further illustrates the importance of mitochondrial dysfunction in the pathogenesis of PrD, with the activation of mitophagy highlighting that mitochondria play an active role in the pathogenesis of PrD. Further study is required to investigate the mechanisms underlying the cellular localisation of PrP^TSE^ to the mitochondrial membrane prior to mitochondrial dysfunction and degradation.

**Fig. 6:**
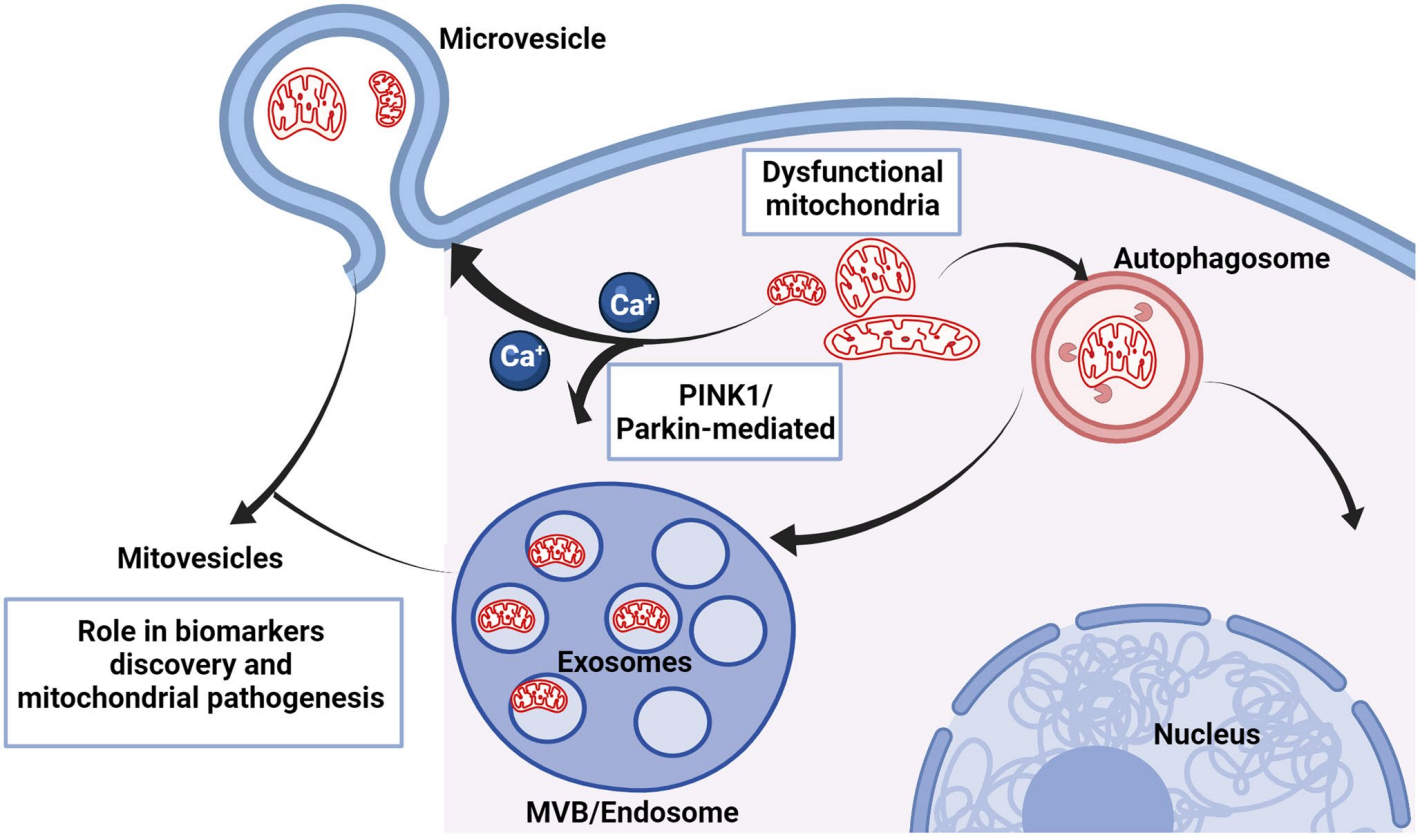
Schematic diagram showing generation of extracellular vesicles (EVs) containing mitochondria (mitovesicles). Dysfunctional or depolarised mitochondria in prion disease evoke an imbalance in calcium ion concentration in the cytosol, consequently inducing the biogenesis of multivesicular bodies and microvesicles. In addition, prion-induced mitophagy with phosphatase and tensin homolog (PTEN)–induced kinase1 (PINK1)/Parkin plays a role in the association of mitochondria to the multivesicular body (MVB) to generate mitovesicles. This figure was generated using Biorender

Interestingly, a novel discovery suggests that EVs may act as potential mitochondrial carriers, including whole mitochondria or mitochondrial fragments. The first clear ultrastructure of highly purified small EVs utilised cryo-electron tomography and discovered EVs with multiple membrane structures (Coleman et al. 2012). Furthermore, the EVs isolated from prion-infected and non-infected GT1-7 cells had different ratios of single membrane to double and triple membrane subpopulations. The discovery of double and triple membrane EVs could suggest that EVs incorporate other membranous organelles during their biogenesis, including mitochondria. In addition, PINK1/parkin are known regulators of intraluminal vesicle (ILV) formation, exosomes release, and the trafficking and association of mitochondria to the endosomal system (McLelland et al. 2016; Song et al. 2016). As observed in PrD, mitochondrial depolarisation causes calcium ion influx in the cytosol, likely impacting EV biogenesis by promoting calcium ion-induced membrane blebbing or MVB fusion to the plasma membrane (Record et al. 2018) (Fig. 6). Cells undergoing mitophagy potentially shuttle the entire mitochondria to microvesicle-sized EVs, a phenomenon observed by several different studies with electron microscopy images (Leermakers et al. 2020; Phinney et al. 2015). Strikingly, one recent study imaged mitochondrial fragments inside small EVs isolated from astrocytes treated with Aβ aggregates (Kim et al. 2020). In the same study, several mitochondrial coding and non-coding RNA biomarkers, including ND1-6, were found elevated in EVs isolated from plasma of AD patients. Furthermore, brain-derived EVs isolated from Down’s syndrome patients contain a different subpopulation of EVs, termed mitovesicles, containing a vast array of mitochondrial constituents (D’Acunzo et al. 2021). Taken together, these observations of mitochondrial dysfunction in PrD and the implications of mitochondrial systems in EV biogenesis highlight a new avenue in the discovery of novel mitochondrial associated EV biomarkers and treatment strategies.

## EVs as prion therapeutics

EVs are both a protective carrier of biological molecules produced by every tissue and a functional body that delivers a signal to the recipient cells. This functional aspect of EVs has become an intense area of research due to the promise of EVs as a potential disease therapeutic strategy. For example, the use of mesenchymal stem cell (MSC)–derived EVs from human placenta intravenously delivered to multiple sclerosis mice demonstrated increased motor abilities, reduced DNA damage and increased myelination (Clark et al. 2019). Another study utilised intra-arterial injection of MSC-derived EVs from human bone marrow to induce an immunomodulatory effect and reduce neuroinflammation in rats with induced focal brain injury (Dabrowska et al. 2019). The ability of EVs injected into the blood to evoke functional effects in the brain across the blood–brain barrier (BBB) makes these an attractive therapeutic candidate for delivering pharmacological treatments capable of targeting PrP^C^ directly to ameliorate pathogenesis of PrD.

In support of this, human neural stem cell (hNSC)–derived EVs injected into the bloodstream ameliorated neuroinflammation and increased cognitive function in immunocompetent mice (Leavitt et al. 2020). Further investigation of the protective effects of NSCs-EVs led to the discovery of the key player, miR-124, in NSCs-EVs cargo responsible for attenuating brain microglial activation. This study suggests that EVs can be engineered for treatment by transfecting cells with miRNA of interest to enrich EV cargo containing miRNA of therapeutic interest. A recent study with this proof of concept demonstrated that astrocyte-derived EVs isolated from cells transfected with miR-29 mimic ameliorated brain ischaemia–reperfusion injury via nuclear factor kappa B/nucleotide-binding domain and leucine-rich repeat-containing family, pyrin domain containing 3 (NF-κB/NLRP3) downregulation in rat brain (Liu et al. 2021). Using suitable candidate miRNA targeting PrP^C^, this technique would be highly relevant in the treatment of PrD as it is both easily scalable and less invasive. A promising candidate recently shown to increase the life expectancy of PrD animal models is the newly developed prion protein reducing antisense oligonucleotides (ASOs) (Minikel et al. 2020). The ability of ASOs downregulating prion protein in this study reversed the disease phenotype and increased survival across various prion strains in a dose-dependent manner. Few challenges of the nucleic acid–based therapeutic intervention are their inefficient biodistribution and susceptibility to breakdown. Therefore, utilising the innate nature of EVs with higher bioavailability and targeted engineering for the functional delivery makes them a highly promising candidate. Furthermore, ASOs are very small molecules of about 10 nucleotides in length which can be engineered in the EVs by electroporation, making the delivery to the brain efficient and less invasive.

## Concluding remarks

EV research is a burgeoning field elucidating the dynamic nature of intercellular communication in the local and distal areas of the body, and has opened a new horizon to decipher the fundamental biological processes in health and disease. Studies exploiting EVs as the biomarker source and their subsequent role in pathogenic and neurotoxic processes aid a better understanding of the complexity of PrD. The gaps in the study of proteomics and lipidomic cargo from the infectious EVs of several prion strains would help to reveal common neurotoxicity-related pathways. Moreover, the functional study of infectious prion EVs in communication with the glial cells can reveal these EVs’ immunomodulatory and inflammatory effects. While secondary lymphoid organs, including the spleen, are indicated as an early accumulation site for prion replication following peripheral prion infection, isolating EVs from these organs for infectivity and cargo assessment would result in better understanding of EVs’ associated roles. Future studies utilising emerging technologies to examine the nanoscale world of EVs will shed more light on this growing field, promoting strain-specific biomarker discovery for disease diagnosis and enabling the engineering of EVs for disease treatment.
